# The impact of repeated temperature cycling on cryopreserved human iPSC viability stems from cytochrome redox state changes

**DOI:** 10.3389/fbioe.2024.1443795

**Published:** 2024-07-30

**Authors:** Jun Okuda, Namiko Watanabe, Tetsuji Nakamura, Kenta Mizushima, Heqi Xi, Yasuaki Kumamoto, Katsumasa Fujita, Masahiro Kino-Oka

**Affiliations:** ^1^ Department of Biotechnology, Graduate School of Engineering, Osaka University, Suita, Japan; ^2^ Research Base for Cell Manufacturability, Osaka University, Suita, Japan; ^3^ R&D Center, Iwatani Corporation, Amagasaki, Japan; ^4^ Department of Applied Physics, Osaka University, Suita, Japan

**Keywords:** transient warming event, human induced pluripotent stem cells, Raman spectroscopy, cold chain, cryopreservation, dimethyl sulfoxide

## Abstract

Human induced pluripotent stem cells (hiPSCs) are an attractive cell source for regenerative medicine. For its widespread use as a starting material, a robust storage and distribution system in the frozen state is necessary. For this system, managing transient warming during storage and transport is essential, but how transient warming affects cells and the mechanisms involved are not yet fully understood. This study examined the influence of temperature cyclings (from −80°C to −150°C) on cryopreserved hiPSCs using a custom-made cryo Raman microscope, flow cytometry, and performance indices to assess viability. Raman spectroscopy indicated the disappearance of mitochondrial cytochrome signals after thawing. A reduction in the mitochondrial membrane potential was detected using flow cytometry. The performance indices indicated a decrease in attachment efficiency with an increase in the number of temperature cycles. This decrease was observed in the temperature cycle range above the glass transition temperature of the cryoprotectant. Raman observations captured an increase in the signal intensity of intracellular dimethyl sulfoxide (DMSO) during temperature cycles. Based on these results, we proposed a schematic illustration for cellular responses to temperature fluctuations, suggesting that temperature fluctuations above the glass-transition temperature trigger the movement of DMSO, leading to cytochrome *c* oxidation, mitochondrial damage, and caspase-mediated cell death. This enhances our understanding of the key events during cryopreservation and informs the development of quality control strategies for hiPSC storage and transport.

## 1 Introduction

Induced pluripotent stem cells (iPSCs) are an important cell source that have been applied to various diseases, such as Parkinson’s disease (Takahashi, 2020), heart failure (Cyranoski, 2018), steroid-resistant acute graft-versus-host disease (Bloor et al., 2020), and retinitis pigmentosa (Kizub et al., 2022) and have been used in numerous therapeutic trials. Although they are primarily used in autologous cell therapies, clinical trials have begun to explore allogeneic human iPSCs (hiPSCs) (Kim et al., 2022). The potential of allogeneic transplantation could substantially broaden the accessibility of cell therapies, as supported by the development of scaled-up manufacturing technologies (Yamamoto and Kino-oka, 2021). For efficient long-term storage, cells are usually stored in the vapor phase of liquid nitrogen (7th Edition NetCord-FACT International Standards for Cord Blood Collection). However, they are vulnerable to transient warming events (TWEs) during transportation and stock management, which can push temperatures beyond safe limits (Pogozhykh et al., 2017; Pasha et al., 2020). Therefore, understanding the effects of TWEs on hiPSCs can optimize cell distribution and enhance cold chain logistics in regenerative medicine.

Studies on TWEs have reported that repeated temperature cycling adversely affects cell viability, and the impact increases with both the frequency and range of the temperature cycles (Vysekantsev et al., 2005; Pogozhykh et al., 2017). Particularly in the range of temperature fluctuations, the involvement of the phase transition, which occurs between the glass phase and the viscous liquid, in the effects on cells has been suggested (Vysekantsev et al., 2005). The glass transition temperature of commonly used cryoprotective agent (CPA), which contains dimethyl sulfoxide (DMSO), has been reported to be around −120°C (Meneghel et al., 2019). Temperature fluctuations between below −150°C in the vapor phase of liquid nitrogen and approximately −80°C during transportation with dry ice and temporary storage in deep freezers can potentially impact cells. The mechanisms underlying the decrease in viability may include a phase transition, which leads to the periodic release of unfrozen bound water fractions (Wolfe et al., 2002) and ice recrystallization, causing an elevation in pressure that results in plastic deformation and ice matrix cracking (Vysekantsev et al., 2005). While studies on human mesenchymal stem cells suggest that apoptosis plays a role in reducing cell viability (Pogozhykh et al., 2017), the mechanisms remain unclear, and no studies have explored the effects of TWEs on hiPSC viability or apoptosis. Another problem is the study method of the TWE, in which thermal sensors are placed in a solution to monitor heating by room-temperature exposure, aiming for a specific target temperature (Angel et al., 2016; Pogozhykh et al., 2017; Xu et al., 2021). However, this method may not ensure precise temperature control due to variations in heat conduction at different sensor locations.

Recent studies proposed the use of Raman signals from cytochrome *c* to assess cell viability during freezing (Yu et al., 2017). Raman spectroscopy, which is beneficial because of its label-free capability to identify and quantitatively recognize spatial information about substances within cells, is effective in uncovering the mechanisms of decreased survival rates. The difficulty in assessing post-thaw damage using Raman spectroscopy lies in the need for low-intensity laser irradiation to minimize cell damage, which requires prolonged acquisition times to achieve an adequate signal-to-noise ratio. These extended acquisition times mean that cells are subjected to the time-dependent effects of exposure to toxic DMSO in the CPA (Kagihiro et al., 2018). This effect is presumed to be accelerated in the presence of freeze-thaw damage; therefore, longer acquisition times should be avoided, especially for cells after thawing. For this solution, slit-scanning Raman microscopy, a high-throughput Raman imaging technique, can drastically shorten the observation time (Kumamoto et al., 2022). To further investigate the involvement of apoptosis, studying the behavior of mitochondrial membrane potential, which affects the initial response, is useful (Kagihiro et al., 2020).

In this study, we employed a custom-made slit-scanning Raman microscope equipped with a sample cryostat to observe the behavior of cytochromes and DMSO in hiPSCs throughout the temperature-cycling process. Furthermore, we assessed the impact of temperature cycling on hiPSC viability and mitochondrial membrane potential using established performance indices and flow cytometry. For precise temperature control, an experiment was conducted using commercially available controlled-rate freezers isolated from ambient-temperature effects.

## 2 Materials and methods

### 2.1 Cell and culture conditions

The hiPSC line 1383D2, sourced from Kyoto University’s Center for iPS Cell Research and Application, was cultured on polystyrene plates coated with laminin-511 E8 (iMatrix-511; Nippi) using StemFit^®^ AK02N medium (Ajinomoto). Sub-culturing was performed as previously described (Yamamoto and Kino-oka, 2021). Briefly, single cells were seeded with 10 μM Rho-associated coiled-coil containing a protein kinase (ROCK) inhibitor (CultureSure Y-27632; Fujifilm Wako Pure Chemical Industries). Initial seeding was fixed at a viable cell density of 7.5 × 10^3^ cells/cm^2^. The cells were incubated at 37°C in a humidified atmosphere containing 5% CO_2_ and the medium was refreshed daily. On day 4, when cells reached 80%–90% confluence, hiPSCs were treated with 5 mM ethylenediaminetetraacetic acid (EDTA) (0.5 mmol/L EDTA/PBS solution; Fujifilm Wako Pure Chemical Industries) and Dulbecco’s phosphate-buffered saline (PBS, Fujifilm Wako Pure Chemical Industries) for 10 min at room-temperature. Cells were dissociated using TrypLE Select^TM^ (Thermo Fisher Scientific) mixed with 10 µM ROCK inhibitor for 7 min at 37°C. After centrifugation, single hiPSCs were resuspended in fresh medium with 10 μM ROCK inhibitor. Viable cells were counted using an automated cell counter (TC20; Bio-Rad Laboratories) after staining with trypan blue.

### 2.2 Suspension of cells with cryopreservation solution

**FIGURE 1:**
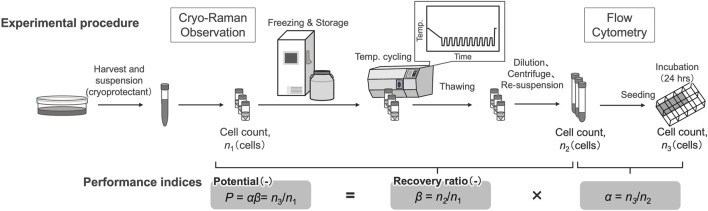
Schematic of the sample preparation and assay for performance indices.

A commercially available CPA, STEM-CELLBANKER GMP grade (Nippon Zenyaku Kogyo), which consists of 10% DMSO, was used in this study. Cells were detached and dissociated for culture maintenance; after centrifugation (180 × *g*, 3 min), the cells were dissociated at a concentration of 1 × 10^6^ cells/mL in the cryopreservation solution, and viable cell concentrations were determined. The hiPSCs were suspended in CPA with 10 µM ROCK inhibitor after being transferred into cryovials (Corning). This solution was used for Raman observations, following the temperature cycling procedure, and for preparing the cryopreserved cell stock (Figure 1).

Cryovials containing hiPSCs suspended in cryopreservation solution with 10 µM ROCK inhibitor were immediately put into a freezing container (CoolCell, Corning) and stored in a −80°C freezer for at least 3 h. Cryovials were transferred to the vapor phase of liquid nitrogen within 24 h and stored until use. These cell stocks were used for temperature cycling. When the cell stock was used for culture, the cells were thawed in a 37°C water bath and diluted with 3 mL fresh medium. After centrifugation (180 × *g*, 3 min), the cells were dissociated at a concentration of 1 × 10^6^ cells/mL in the medium and cultured.

### 2.3 Temperature cycling procedure

This procedure was performed in a commercial controlled-rate freezer (CryoMed controlled-rate freezer; Thermo Fisher Scientific). To investigate the impact of the number of temperature cycles, temperature range were set from −150.0°C to −80.0°C, with cycles counts of 10, 20, 30, 50, and 70. The effects of temperature range were studied over four ranges: from −170.0°C to −150.0°C, from −150.0°C to −130.0°C, from −150.0°C to −115.0°C, and from −115.0°C to −80.0°C, with 30 cycles each. Experimental cell stock was positioned in the chamber of the controlled-rate freezer, and maintained at −150.0°C; then temperature cyclings were performed as per the designated program. After completion of the program, the cells were transferred to the liquid nitrogen vapor phase for their intended use. All temperature programs were set to a warming rate of 4.0°C/min and cooling rate of 40.0°C/min; after reaching the target temperature, the temperature was maintained for 10 min before moving to the next program. An example of such a program is presented below.

1. Hold @ −150.0°C 10 min
2. 4.0°C/min to −80.0°C
3. Hold @ −80.0°C 10 min
4. −40.0°C/min to −150.0°C
5. Hold @ −150.0°C 10 min
6. Repeat steps 2–5 for up to 69 times

### 2.4 Slit-scanning Raman microscope equipped with a sample cryostat

**FIGURE 2:**
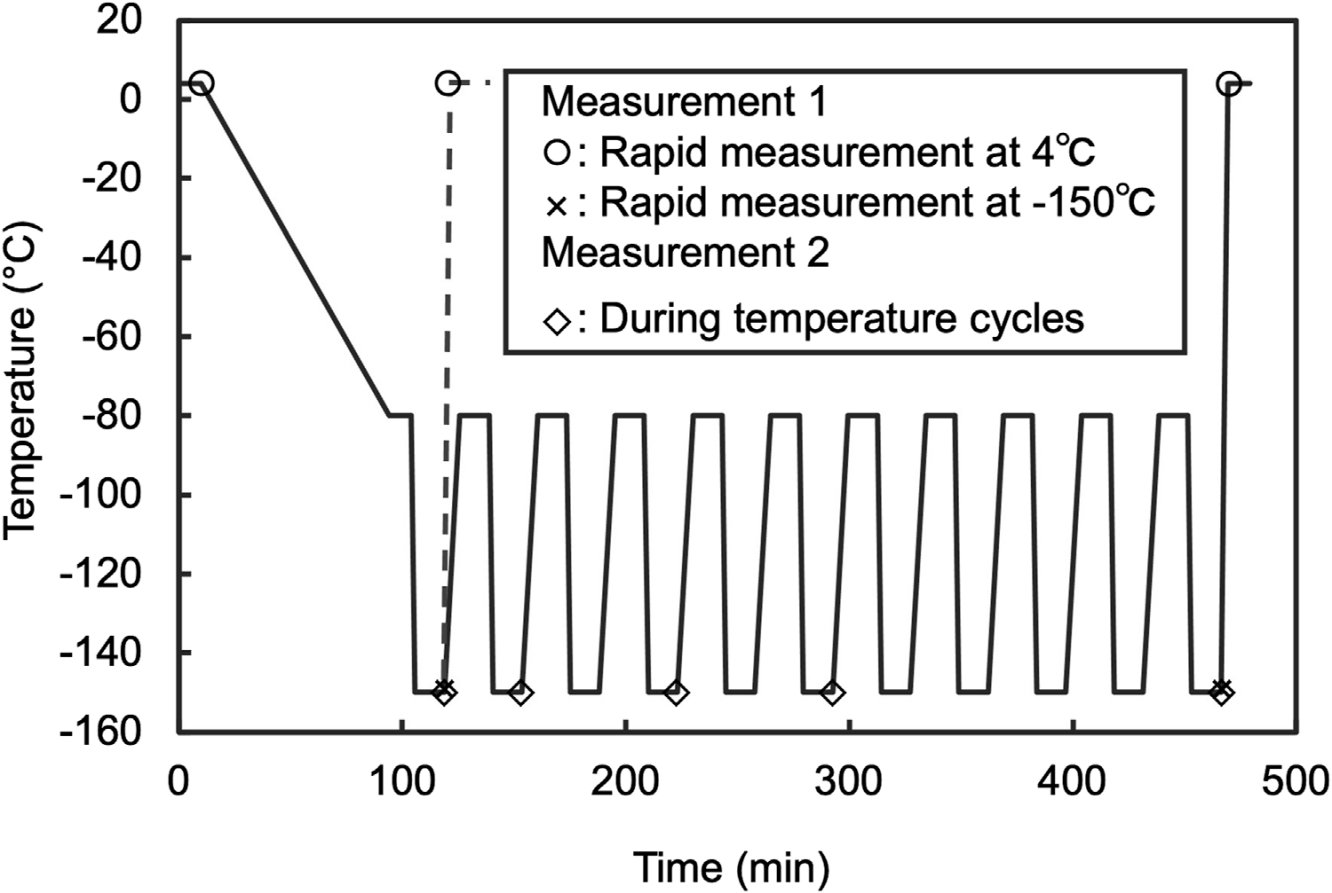
Thermal program of sample cryostat applied to Raman observation. For measurement 1, the rate of warming to 4°C at the end of the program was 40°C/min. Measurement was started as soon as the temperature reached 4°C. In addition, the measurement performed after 10 cycles was designated as Temp. cyclings +. Furthermore, for comparison to before freezing, the measurement that was performed on the stage after cooled to 4°C and held for 10 min was designated as Freezing −. Measurement 2 protocol was adjusted to include a 10 min hold at −150°C before starting measurements, then proceeded to the next step after completion. Measurements were conducted at the 1st, 3rd, 5th, and 10th temperature cycles. Prior to the start of the cycles, a control measurement was performed after a 10 min wait from the first instance of reaching −150°C.

All measurements were conducted using a slit-scanning Raman microscope equipped with a sample cryostat. For cell imaging, a previously reported system (Mizushima et al., 2023) featuring a sample cryostat (“THMS600, Linkam”; customized for an inverted microscope) attached to the sample stages of a home-built Raman microscope system was utilized. A coverslip was then placed on the sample holder. Further information on the optical setup is provided in (Kumamoto et al., 2022). Briefly, a 532 nm laser with continuous-wave oscillation (Millennia eV, Spectra Physics or Verdi 532 nm, Coherent) was used for Raman excitation. The laser intensity at the sample was set at 3.0 mW/μm^2^. This intensity was determined after confirming that there were no observable changes, such as blebbing or membrane disruption, in the cell morphology after five repeated measurements. The laser beam was focused into a linear shape using a cylindrical lens and on the sample located on an inverted microscope (Ti-E, Nikon) equipped with a 60 × /0.95 NA dry objective lens (CFI Plan Apo Lambda 60XC, Nikon). The Raman scattering light generated at the sample under line illumination was collected by the same objective lens in a back scattering geometry, filtered with a longwave-pass edge filter, and refocused at the entrance slit (60 μm) of a spectrophotometer (CLP-300 or MK-300, Bunkoukeiki) equipped with a grating of 600 lines/mm. The one-dimensional distributions of the Raman spectra of the samples irradiated by line illumination were recorded using a cooled CCD camera (PIXIS, 2048 B or PIXIS 400BReXcelon, Teledyne Princeton Instruments). A galvanometer mirror was used to scan the sample and acquire a two-dimensional Raman hyperspectral image. The scanning pitch was set at 1.0 μm. A focus drift system equipped with a microscope (Nikon PFS) was used to stabilize the laser focus position during Raman imaging. The measurements during temperature cycling were performed under the conditions shown in Figure 2. For the post-thaw rapid measurements, the following conditions were applied (Measurement 1, Figure 2): laser intensity at 3.0 mW/μm^2^, spectrophotometer entrance slit width at 55 μm, grating at 300 lines/mm, and scanning pitch at 1.0 μm. The total time required to acquire a Raman hyperspectral image, including the exposure and data readout for all lines, was approximately 80 s. The wavenumber axis of the Raman spectra was corrected using the ethanol Raman bands at 434, 884, 1,454, and 2,930 cm^−1^ as references.

The cell suspension sample was placed on a cover glass with a 20 μm spacer. Another cover glass was then placed on top to sandwich the sample. This assembly was mounted onto the sample holder of a Linkam stage for observation. Cell suspension temperatures were alternately cycled 10 times between −80.0°C and −150.0°C using the cryostat, with heating and cooling rates set at 4.0°C/min and −40.0°C/min, respectively. The process was controlled and measurements were performed according to the temperature profile presented below and in Figure 2.

1. Hold @ 4.0°C 10 min
2. −1.0°C/min to −80.0°C
3. Hold @ −80.0°C 10 min
4. −40.0°C/min to −150.0°C
5. Hold @ −150.0°C 10 min
6. 4.0°C/min to −80.0°C
7. Repeat steps 3–6 for 9 times
8. 50.0°C/min to 4.0°C
9. Hold @ 4.0°C

### 2.5 Raman data processing

**FIGURE 3:**
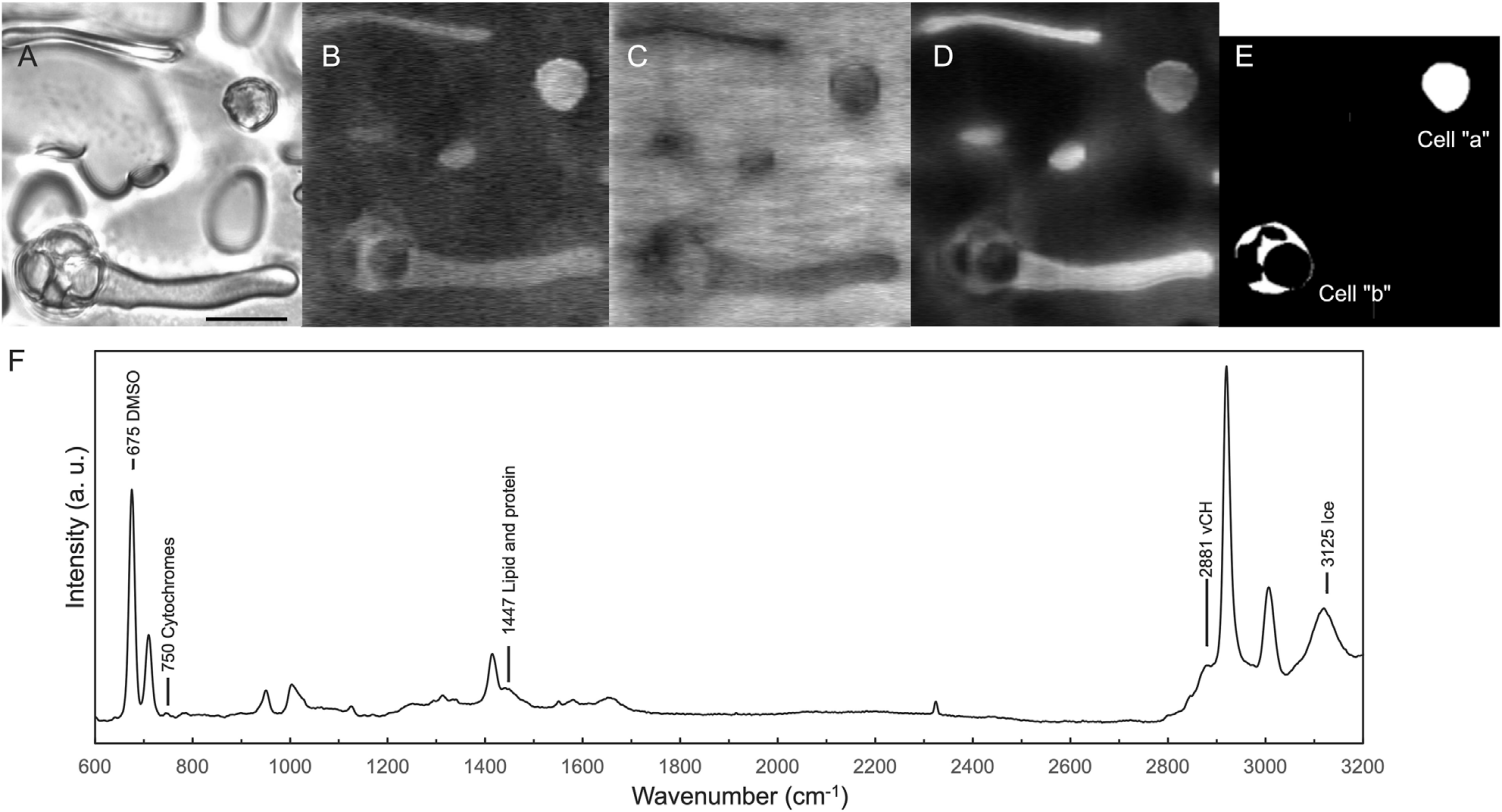
Area determination and peak area normalization applied for data analysis. Bright-field image acquired at −150°C **(A)**, Raman image created from the intensity of the νCH signal at 2,881 cm^−1^ **(B)**. Images that identify the locations of ice and viscous liquid were created from the ice νOH signal at 3,125 cm^−1^ **(C)** and the DMSO CS stretching signal at 673 cm^−1^ **(D)** (Li et al., 2018). Based on **(A)** and **(B)**, the cellular regions were visually selected as cell “a” and cell “b” **(E)**, and average spectra were extracted from each region. The peak areas of DMSO and cytochromes from the average spectra were normalized to the peak area of δCH signal at 1,447 cm^−1^ **(F)**. Scale bar, 20 μm.

The Raman hyperspectral imaging data were processed using a custom analytical platform constructed in MATLAB (R2023b, MathWorks). Cosmic rays were removed using median filtering. The background level by CCD bias was corrected to 0. The spectral ranges in Table 1, selected based on the assigned wavenumber, were utilized for the peak area calculation and Raman image reconstruction. The baseline was defined as a straight line connecting the two points specified by these ranges. The peak area was then determined by numerical integration of the region between the baseline and the peak. The cellular regions for the quantification of the cell-derived signal were determined based on the bright-field and Raman images obtained from the νCH signal at 2,881 cm^−1^ (Figures 3A, B), (Czamara et al., 2015). Furthermore, images identifying the locations of ice and viscous liquid were created from the ice νOH signal at 3,125 cm^−1^ and the DMSO CS stretching signal at 673 cm^−1^ (Figures 3C, D). We used the peak area of δCH (1,447 cm^−1^) assignable to lipid and protein (Czamara et al., 2015; Okotrub and Surovtsev, 2015; Ikemoto et al., 2021) to normalize the peak area of the substance to be quantified in the average spectrum acquired from the cellular region (Figure 3E). The normalized value was calculated by dividing the peak area of the target substances (such as cytochromes and DMSO) by the peak area of δCH (Figure 3F) as the signal at 1,447 cm^−1^ was obtained uniformly in the cells (Supplementary Figure S1) and the wavenumber range did not contain the resonance signals of the cytochromes (Okotrub and Surovtsev, 2015). The DMSO Raman images were created using DMSO signal intensities, which represent the ratio of the DMSO peak intensity at 675 cm^−1^ to the δCH peak intensity at 1,447 cm^−1^. Plots for each band were fitted to a linear function using a custom-made Python program.

**TABLE 1:** Spectral range selection of Raman data analysis.

Substance	Wavenumber (cm^−1^)	Assignments
DMSO	663.1–695.4	Symmetric CS stretching Li et al. (2018)
Lipid and protein	1429.0–1503.8	δ (CH) Ikemoto et al. (2021)
Cytochromes	750.7–765.0	Pyrrole breathing ν15 Okotrub and Surovtsev (2015)

### 2.6 Definition of performance indices and assay methods

Variation of cell viability after temperature cyclings was evaluated using established performance indices, recovery ratio (*β*) and attachment efficiency (*α*) (Kagihiro et al., 2018). Before freezing, the viable cells were counted using an automated cell counter following trypan blue staining. The number of viable cells obtained was *n*_1_ for subsequent calculations. After cell counting, cell suspensions were dispensed into cryotubes and placed in a freezing container. After storing in a −80°C freezer for at least 3 h, cryotubes were transferred to liquid nitrogen storage within 24 h. The recovery ratio (*β*) is defined as the ratio of viable cells after freezing and thawing compared to the viable cells before freezing, and is represented by Eq. 1.

$$\beta =\frac{n_{2}}{n_{1}}$$ (1)

where *n*_2_ is the total number of viable cells after freezing and thawing, as measured using the following procedure: after thawing in a 37°C water bath, the cells were diluted to 7 times their volume in fresh medium. The cells were centrifuged (180 × *g*, 3 min), resuspended in the medium, and viable cells were counted using an automated cell counter after trypan blue staining.

To estimate attachment efficiency of cells after freezing and thawing, the thawed cells were seeded at a density of 2.5 × 10^3^ cells/cm^2^ into eight-well plates coated with laminin-511 E8 fragments in which the medium contained 10 μM ROCK inhibitor and incubated at 37°C in a humidified atmosphere with 5% CO_2_ for 24 h. The attachment efficiency (*α*) is defined as the ratio of the total number of attached cells relative to the seeded number of viable cells after freezing and thawing, given in Eq. 2.

$$\alpha =\frac{n_{3}}{n_{2}}$$ (2)

where *n*_3_ is the total number of viable cells on the culture plate surface after 24 h, when *n*_2_ of the viable cells were seeded after freezing and thawing. In this study, cells that attached to the surface of the culture plate were counted using a phase-contrast microscope at × 10 magnification.

The potential of viable cells (*P*) was defined as the ratio of the number of total viable cells on the culture plate surface after 24 h of incubation to *n*_1_, as shown in Eq. 3

$$P=\frac{n_{3}}{n_{1}}$$ (3)

### 2.7 Flow cytometric analyses

To assess the accumulation of the mitochondrial membrane potential (*ΔΨ*_m_) in the cells after temperature cyclings, flow cytometric analyses were conducted. In this analysis, the medium containing 10 μM ROCK inhibitor was used. The cells were thawed and resuspended in the medium. After incubation at 37°C for 2 h, the suspension was diluted to 3.3 × 10^5^ cells/mL with the medium. The cell suspensions were incubated with 200 nM of active mitochondrial specific dye (tetramethylrhodamine ethyl ester (TMRE); Abcam) at 37°C for 15 min, then 5 nM of dead cell stain (SYTOX Red (SR); Thermo Fisher Scientific) was added and incubated at 37°C for 15 min. The fluorescence intensity of the stained cells was detected using a flow cytometry system (CyFlow Cube 6; Sysmex Partec) at excitation/emission wavelength of 488 nm/536 nm for TMRE and 638 nm/675 nm for SR. Cellular debris was excluded based on the properties of the forward- and side-scattered light. In total, more than 5 × 10^3^ events were analyzed using flow cytometry and data analysis software (FCS Express 6 software; *De Novo* Software).

### 2.8 Differential scanning calorimetry (DSC)

DSC experiments on CPA were performed using a Differential Scanning Calorimeter (DSC8000, Perkin Elmer). Temperature calibration was performed using cyclohexane (crystal-crystal transition at −87.1°C, melting point at 6.7°C), Indium (melting point at 156.6°C). Approximately 25 mg of CPA (STEM- CELLBANKER GMP grade, Nippon Zenyaku Kogyo) were placed in each of 50 μL Perkin Elmer DSC aluminum pans and sealed. The DSC run for each sample began at 20.0°C. Samples were cooled to −150.0°C at a rate of −10.0°C/min and subsequently heated to 20.0°C at a rate of +10.0°C/min. An empty pan was used as a reference. The results were obtained from three separate cooling-warming cycles.

The glass transition temperatures (*T*_g_, °C) were determined by plotting the first derivative of the heat flow over time against the temperature from the heat-flow data recorded during warming. The data were processed using 500-point smoothing on a Savitzky-Golay filter. The temperature at the peak top of the typical local maximum, which represents the glass transition temperature appearing near −120°C (Meneghel et al., 2019), was defined as *T*_g_.

### 2.9 Statistics

One-way ANOVA followed by Tukey’s test was used to evaluate differences between groups, and differences between means were considered statistically significant at *p* < 0.05.

## 3 Results

### 3.1 Cytochromes signal detection after thawing

**FIGURE 4:**
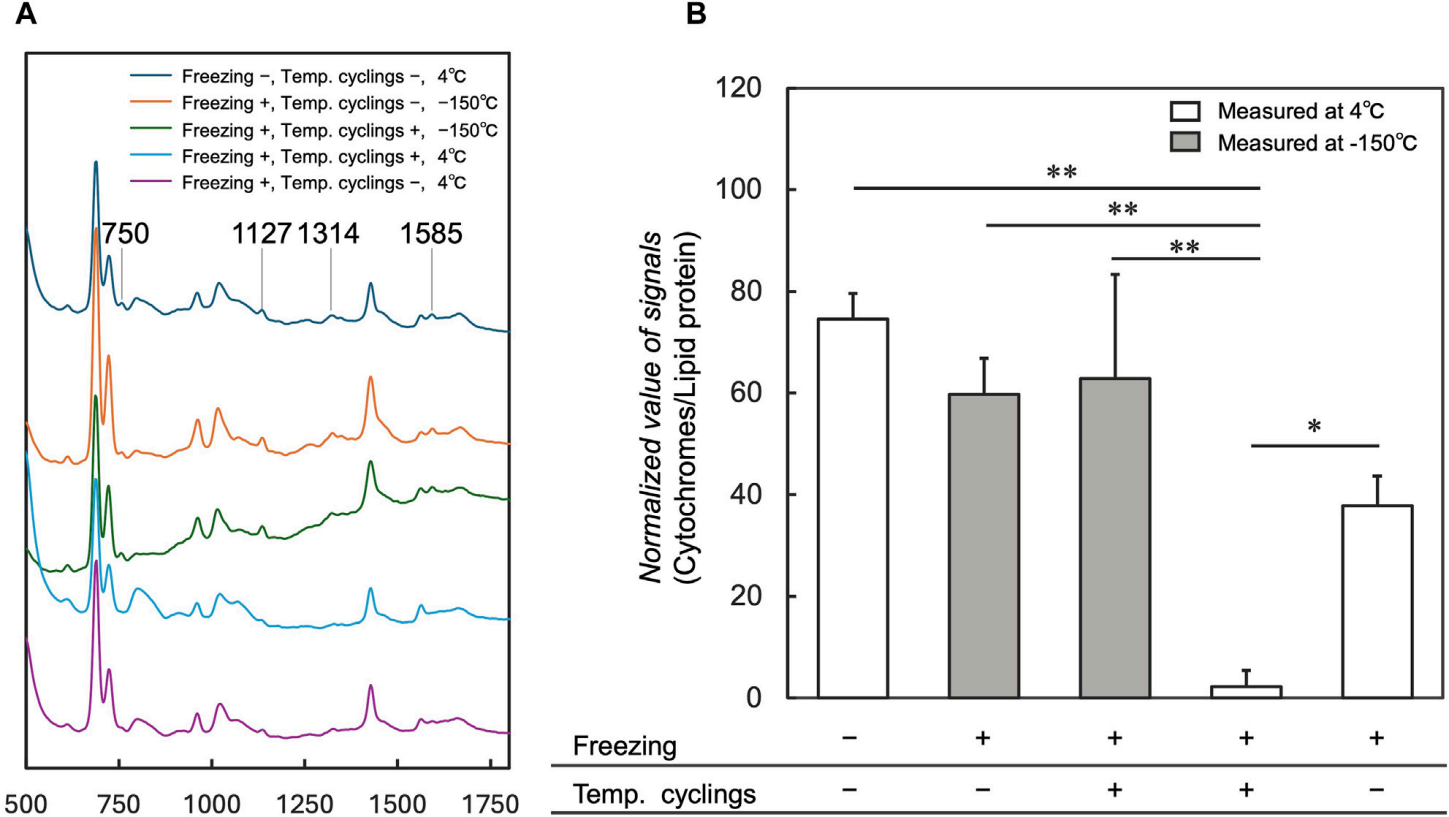
Comparison of average spectra and normalized values of the cytochromes signals for the measurements at 4°C and −150°C with and without temperature cycling. Average spectra of cell regions are shown in **(A)**, and Raman signals at 750, 1,127, 1,314, and 1,585 cm^−1^ are assigned to the vibrational modes of cytochrome *c*. Normalized values of the signals are shown in **(B)**. “-” indicates the absence of temperature cycling and freezing, whereas “+” indicates the presence of these processes. Data is shown as the mean ± standard deviation of 3 experiments. *p < 0.05, **p < 0.01; Tukey’s test.

To investigate the influence of 10 temperature cycles on cytochromes that trigger cell death, cytochromes signals were measured in cells after freezing, temperature cycling, and thawing at 4°C using the cryo-Raman system, compared with those without temperature cycling. Average spectra acquired from cell regions under conditions without freezing and temperature cyclings showed cytochromes signals at 750, 1,127, 1,314, and 1,585 cm^−1^ (Figure 4A “Freezing −, Temp. cyclings −, 4°C”) (Okada et al., 2012). These signals were markedly reduced under the condition with temperature cyclings at 4°C (Figure 4A “Freezing +, Temp. cyclings +, 4°C”) when compared to the spectra obtained under conditions without freezing and temperature cyclings (Figure 4A “Freezing −, Temp. cyclings −, 4°C”). The normalized signal of cytochromes significantly decreased in samples with temperature cyclings (Figure 4B “Freezing +, Temp. cyclings +, 4°C”) compared to control; without temperature cyclings (Figure 4B “Freezing +, Temp. cyclings −, 4°C”). We also measured samples with and without temperature cycling at −150°C (Figure 4A “−150°C”; Figure 4B “Measured at −150°C”). The average spectra acquired from cell regions under both conditions showed cytochromes signals at 750, 1,127, 1,314, and 1,585 cm^−1^ (Figure 4A “−150°C”). The normalized signal of cytochromes under the condition with temperature cyclings at 4°C (Figure 4A “Freezing +, Temp. cyclings +, 4°C”) was lower than signals under these conditions.

### 3.2 Raman imaging of DMSO across temperature cycles

**FIGURE 5:**
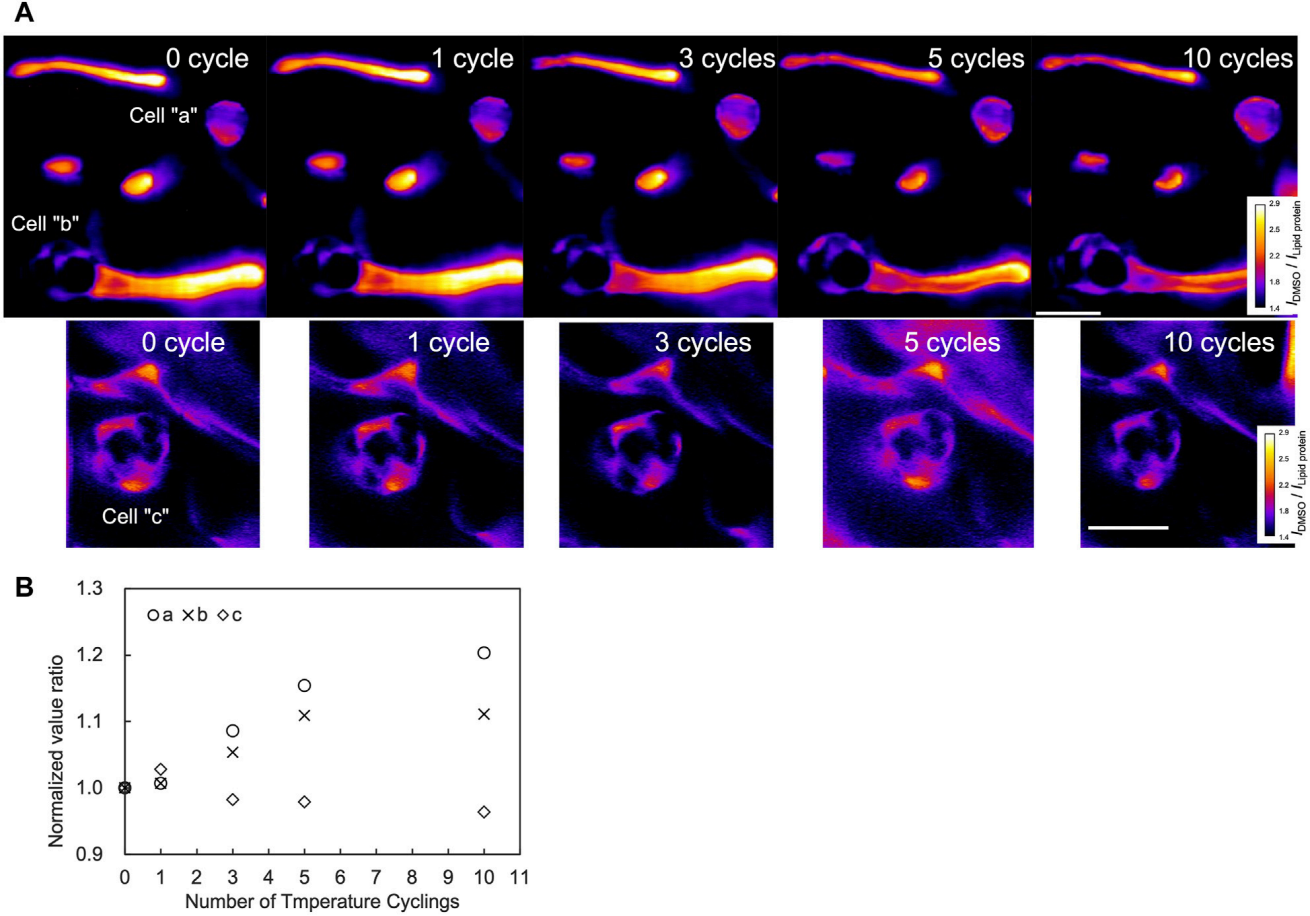
Raman images and signal quantification through temperature cycles. Measurements were performed on a region containing three cells as shown in **(A)**. The color bar illustrates DMSO signal intensities. Scale bar, 20 μm. Normalized signal at each number of cycles were further normalized by the value of the 0th cycle **(B)**.

To investigate the DMSO dynamics associated with temperature cycling, Raman imaging was performed for the area including the cells over the cycles. From the Raman images, the areas within the cells exhibited higher intensity after 5 and 10 temperature cycles compared to the 0 cycle (Figure 5A, cell “a” and cell “b”). The ratio of the normalized DMSO signal relative to the zero cycle increased up to the 5th cycles for both cells, as shown in Figure 5B. Notably, cell “b” maintained a signal ratio of 1.1 after the 5th cycle, whereas cell “a” showed an increasing trend, to a ratio of 1.2 up to the 10th cycle (Figure 5B). In contrast, cell “c” did not show a significant increase over the cycles.

### 3.3 Detection of Δ*Ψ*_m_ changes by temperature cycling

To determine the effect of temperature cycling on mitochondria, *ΔΨ*_m_, as well as plasma membrane integrity, were evaluated with TMRE and SR via flow cytometer. The percentage of TMRE-, SR- and TMRE-, SR+ indicating reduced *ΔΨ*_m_ increased with 30 or more temperature cycles (Table 2). The percentages of TMRE+ and SR-, indicating healthy cells, decreased to 67.2% after 30 temperature cycles and did not change with increased number of temperature cycles. In contrast, the percentages of TMRE+ and SR+ cells, which indicate the loss of plasma membrane integrity, were low after 30 or more temperature cycles.

**TABLE 2:** Flow cytometry with TMRE and SR for analysis of *ΔΨ*_m_ and plasma membrane integrity of hiPSCs after temperature cyclings.

		TMRE+, SR-	TMRE-, SR-	TMRE-, SR+	TMRE+, SR+
Control (no cycle)		78.5 ± 7.7	5.3 ± 3.7	7.6 ± 4.8	8.6 ± 4.7
Temp. cycles from −150°C to −80°C	10 cycles	76.1 ± 3.9	3.9 ± 1.3	5.2 ± 1.4	13.8 ± 3.8^††^
	30 cycles	67.2 ± 3.3*	7.7 ± 0.7	21.3 ± 2.7**^,††^	3.8 ± 0.4^††^
	50 cycles	67.7 ± 3.3*	11.2 ± 2.1*^,††^	16.4 ± 2.0*^,††^	3.4 ± 0.7^††^
	70 cycles	68.6 ± 5.1*	11.5 ± 0.5*^,††^	17.5 ± 4.5**^,††^	2.4 ± 0.3^††^

TMRE+ exhibits high *ΔΨ*_m_, and TMRE- exhibits low *ΔΨ*_m_. SR- exhibits high plasma membrane integrity, SR+ exhibits low plasma membrane integrity. Data are shown as the mean ± standard deviation of four experiments. *p < 0.05, **p < 0.01 (versus no cycle); ^†^ *p* < 0.05, ^††^ *p* < 0.01 (versus 10 cycles); Tukey’s test.

### 3.4 Evaluating performance indices under temperature cyclings

**FIGURE 6:**
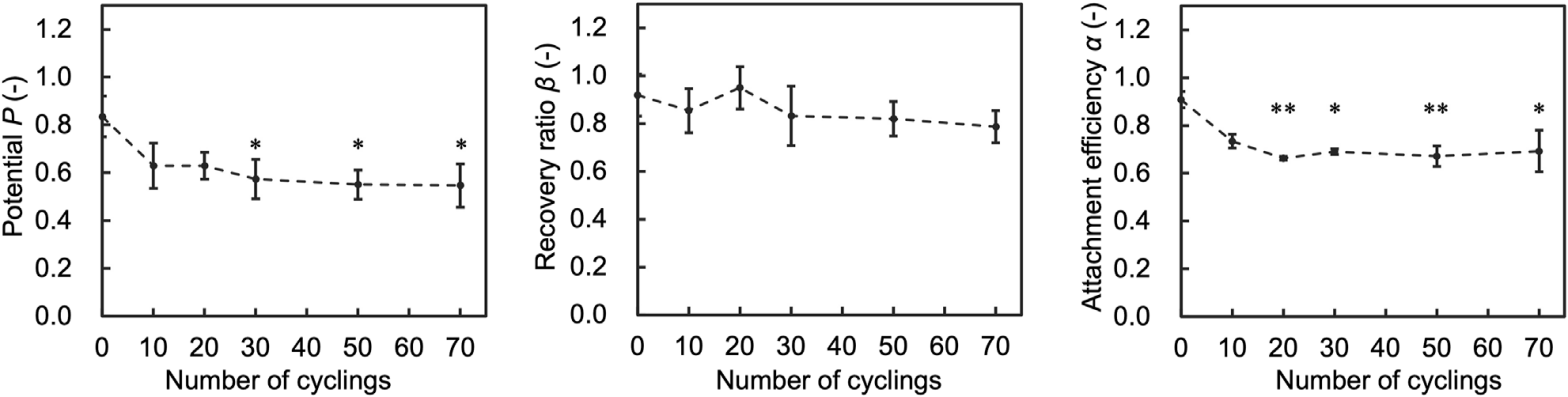
Results of assessment on the number of temperature cycles affecting attachment efficiency (*α*), recovery ratio (*β*), and Potential (*P*). Data are shown as the mean ± standard deviation of 4 experiments. *p < 0.05, **p < 0.01; Turkey’s test.

To assess the impact of number of temperature cycles on the performance indices (Attachment efficiency (*α*), recovery ratio (*β*), and Potential (*P*)), we assessed cells after experiencing 10, 20, 30, 50, and 70 temperature cycles. The results showed that *α* decreased as the number of temperature cycles increased and did not change significantly after 20 cycles (Figure 6). *P* also decreased accordingly to *α*. *β* did not change significantly with the number of cycles.

**FIGURE 7:**
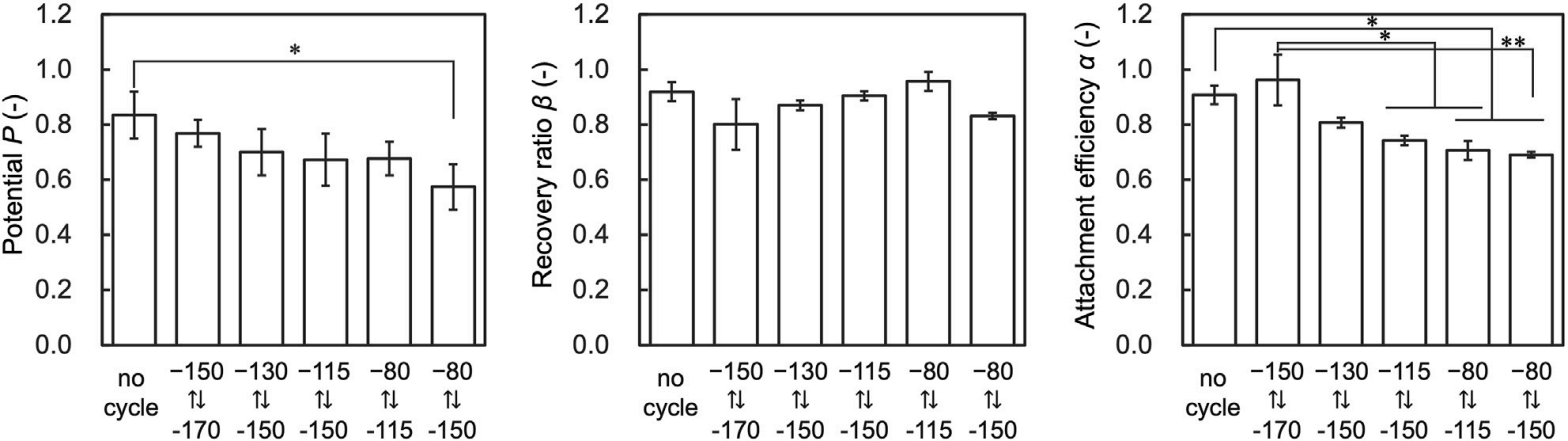
Results of assessment on the temperature range affecting attachment efficiency (*α*), recovery ratio (*β*), and Potential (*P*). Data are shown as the mean ± standard deviation of 4 experiments for hiPSCs. *p < 0.05, **p < 0.01; Turkey’s test.

To further investigate the effect of temperature cycling on performance indices, we evaluated cells that fluctuated 30 times in each temperature range. The results showed that *α* decreased with the temperature range of from −150.0 to −80.0°C and −150.0 to −115.0 compared to the control, and *P* decreased with the temperature range of −150.0 to −80.0 compared to the control (Figure 7). *β* showed no significant deviation from control conditions under any tested conditions.

### 3.5 DSC measurement to evaluate *T*_g_ of CPA

From the trace of heat transfer during heating, a shift around −120°C was observed. A peak was confirmed in the first derivative of the heat transfer, which was defined as *T*_g_ (see Supplementary Figure S2). The results of three independent measurements of cooling and heating regarding this peak top were −116.6°C ± 0.8 (mean ± standard deviation).

## 4 Discussion

Although several studies have reported reduced cell viability due to temperature fluctuations, the underlying mechanisms behind this phenomenon remain elusive. Our study revealed a sharp decline in cytochromes signals following freezing and temperature cyclings at 4°C. The observed signal reductions aligned with the changes attributed to cytochrome *c* oxidation (Okada et al., 2012). While bands for lipid and protein (1,447 cm^−1^) were observed (Figure 4A), and it was reasonable to assume that their levels did not change, the reduction in signals was considered to correspond to cytochromes oxidation. The reference measurements for samples with and without temperature cycling at −150°C showed that the cytochromes signals were detected in both cases, with no significant change in their levels due to temperature cycling. Considering the presence of the cytochromes signal at −150°C after the temperature cyclings, the signal must have decreased before the sample was thawed to 4°C. These findings suggested that the signal decrease in cytochromes caused by temperature cycling could be attributed to post-thaw cytochrome *c* oxidation.

Notably, one of the earliest events in apoptosis triggered by death receptors is the rapid release of cytochrome *c* within 5 min (Goldstein et al., 2000). However, our experiments indicated that considering the necessity of water molecules for the movement of substances and enzymatic reactions (Klibanov, 2001), the disappearance of signals occurred immediately after thawing, assuming that the reactions begin once ice melts. A past study showed that the extracellular ice starts melting from −60°C when cryopreserved cells with 10% DMSO-based CPA are warmed from −100°C (Pogozhykh et al., 2017), similar to that used in this study. Applying this temperature, it can be inferred that the disappearance of signals occurred within 3 min after the beginning of extracellular ice melting, as measurements at 4°C took 80 s and the warming time from −60°C to 4°C was 96 s. This rapid response led us to deduce that oxidation preceded the release of cytochrome *c*. This oxidation process was supposed to peroxidize cardiolipin, facilitating the release of cytochrome *c* from the mitochondria (Brown and Borutaite, 2008; Okotrub and Surovtsev, 2015; Kitt et al., 2017) and subsequently causing a loss of mitochondrial membrane potential (Yuan et al., 2014), corroborated by the decrease in TMRE fluorescence intensity in flow cytometry approximately 2 h post-thaw. The release of cytochrome *c* triggers apoptosome formation, resulting in caspase activation (Brown and Borutaite, 2008; Kole et al., 2011).

Further analysis of the performance indices under temperature cycling showed no significant changes in the recovery ratio of hiPSC viability. However, a notable reduction in attachment efficiency was observed, indicating a delayed effect of temperature cycling on cell viability. The decline in attachment efficiency was particularly evident during cycles passing −115°C, a temperature range identified as above the glass transition temperature (*T*_g_) of −116.6°C, as measured by DSC (Supplementary Figure S2), aligning with the hypothesis that degradation occurs with cyclings exceeding *T*_g_ (Vysekantsev et al., 2005; Mrowiec et al., 2012).

Raman observations successfully captured the movement of DMSO caused by temperature cycling. DMSO-based CPA is concentrated between ice crystals and maintains a high-viscosity state exceeding 10^12^ Pa s when maintained at temperatures below its *T*_g_ (Hubel et al., 2014; Fonseca et al., 2016; Meneghel et al., 2019). However, upon exceeding *T*_g_, the viscosity decreased to 100 mPa s (Erickson, 2009; Meneghel et al., 2019). This rapid decrease in viscosity leads to a substantial increase in fluidity. Moreover, the liquid phase expands substantially more than the surrounding pure ice because of its higher thermal expansion coefficient (Rabin and Bell, 2003a; Rabin and Bell, 2003b; Xu et al., 2007), which may accelerate the movement of the liquid phase. Given that DMSO diffuses through cell membranes (Edashige, 2017), these thermomechanical dynamics may enable it to permeate cell membranes, resulting in an increased DMSO signal detected in some cells using Raman spectroscopy. Exposure to DMSO enhances the activation of cytochrome *c* oxidase, which causes subsequent oxidation of cytochrome *c* (Desai et al., 1988). The observed increase in the DMSO signal suggests the augmented presence of DMSO within the cells, potentially triggering a cascade starting with the oxidation of cytochromes. This process could lead to a decrease in the mitochondrial membrane potential and subsequent cell death after thawing. This does not negate the hypothesis that cell damage may results from the release of unfrozen bound water fractions (Wolfe et al., 2002; Pogozhykh et al., 2017) or ice recrystallization (Vysekantsev et al., 2005). Consequently, cell damage from temperature cycling resulted in delayed cell death as a reduction in attachment efficiency, suggesting that the effects were not primarily due to lethal damage to the cell membrane, but were related to mitochondrial dysfunction. The most notable reduction in attachment efficiency was observed in the 10th cycle, with no further changes beyond 20 cycles, which may be related to the disappearance of cytochromes signal by the 10th cycle. The shape of the cells can be affected by the ROCK inhibitor used in the medium during the experiment (Harbom et al., 2019; Münsterberg et al., 2021), and the observed behavior occurred under these conditions.

**FIGURE 8:**
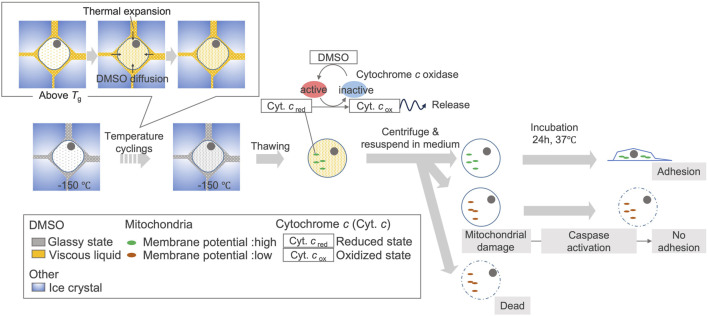
Proposed schematic illustration of cellular response to temperature fluctuations in hiPSCs due to TWEs. Due to temperature cycling, the rise in temperature above the glass transition temperature of CPA causes the extracellular concentrated DMSO to become viscous liquid. Along with thermal expansion, this leads to the migration of DMSO into some cells. Intracellular DMSO can activate cytochrome *c* oxidase after thawing, leading to the oxidation of cytochrome *c*. When cytochrome *c* is oxidized, some cells experience mitochondrial membrane potential disruption, which may prevent cell adhesion and eventually induce cell death. The relationship between DMSO migration and cytochrome *c* oxidation is based on a research (Desai et al., 1988). The relationship of cytochrome *c* oxidation, cytochrome *c* release, mitochondrial membrane potential disruption is also based on past studies (Goldstein et al., 2000; Brown and Borutaite, 2008).

**FIGURE 9:**
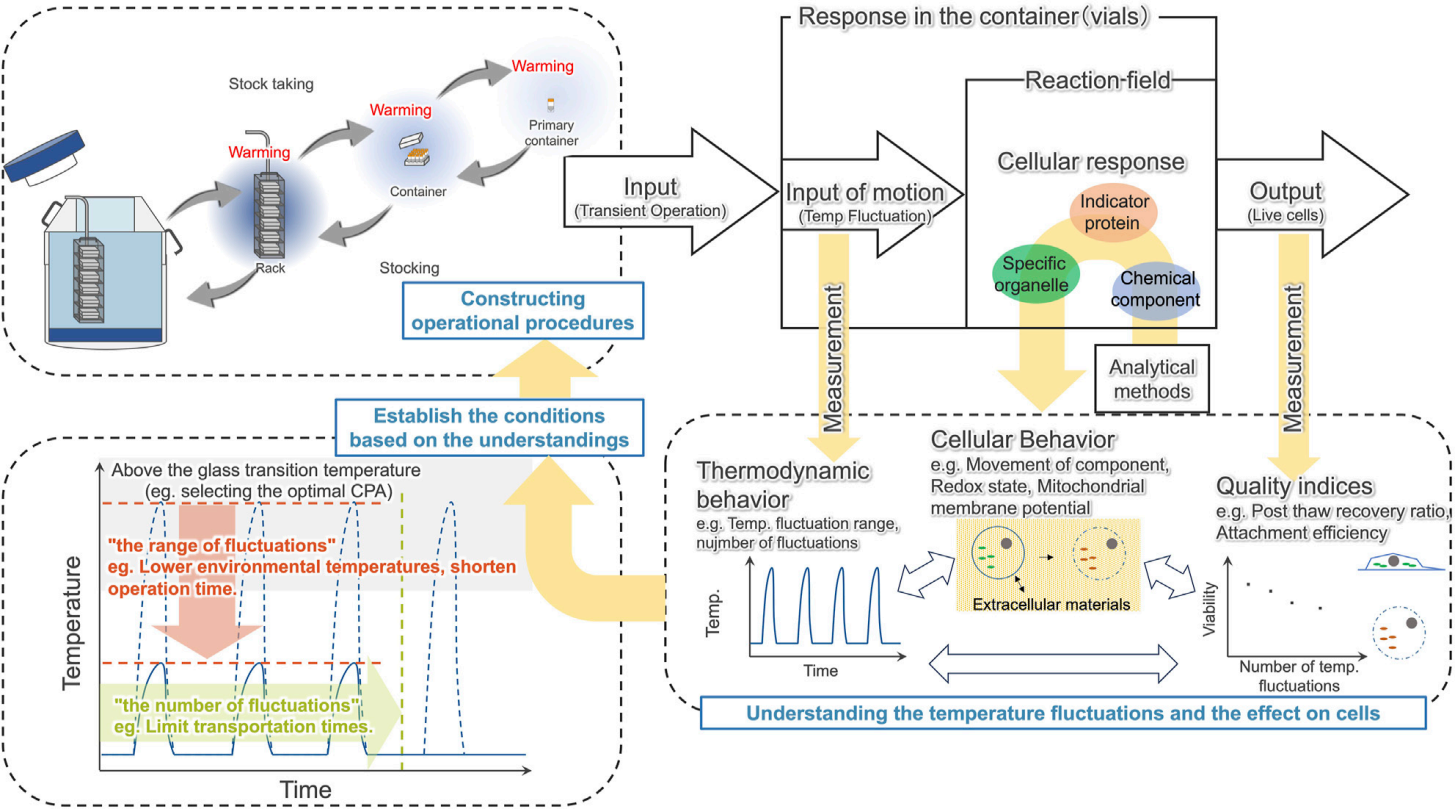
Constructing operational procedures based on understanding the temperature fluctuations caused by transient handling procedures and understanding the effect on cells.

In this study, we captured the events that can lead to decreased viability of hiPSCs due to temperature cycling, and found that the disappearance of the cytochromes signal starts immediately after thawing. Based on this, we proposed a schematic illustration of the cellular response to temperature fluctuations (Figure 8). To maintain cell quality against such degradation, we suggest understanding the temperature fluctuations caused by transient handling procedures and effect on cells, then constructing operational procedures based on this understanding of how temperature fluctuations affect cells (Figure 9).

## Data Availability

The original contributions presented in the study are included in the article/Supplementary Material, further inquiries can be directed to the corresponding author.

## Supplementary material

SUPPLEMENTARY FIGURE S1 Bright-field image acquired at −150°C **(A)** and Raman image created from the intensity of the δCH signal at 1,447 cm^−1^ **(B)**.

SUPPLEMENTARY FIGURE S2 A typical DSC trace of CPA used for the study during warming, and the first-order derivative.
