# Supplementary figures and images for: The impact of repeated temperature cycling on cryopreserved human iPSC viability stems from cytochrome redox state changes
Source: Front Bioeng Biotechnol. 2024 Jul 30;12:1443795. doi: 10.3389/fbioe.2024.1443795 (PMC11319289; doi:10.3389/fbioe.2024.1443795)

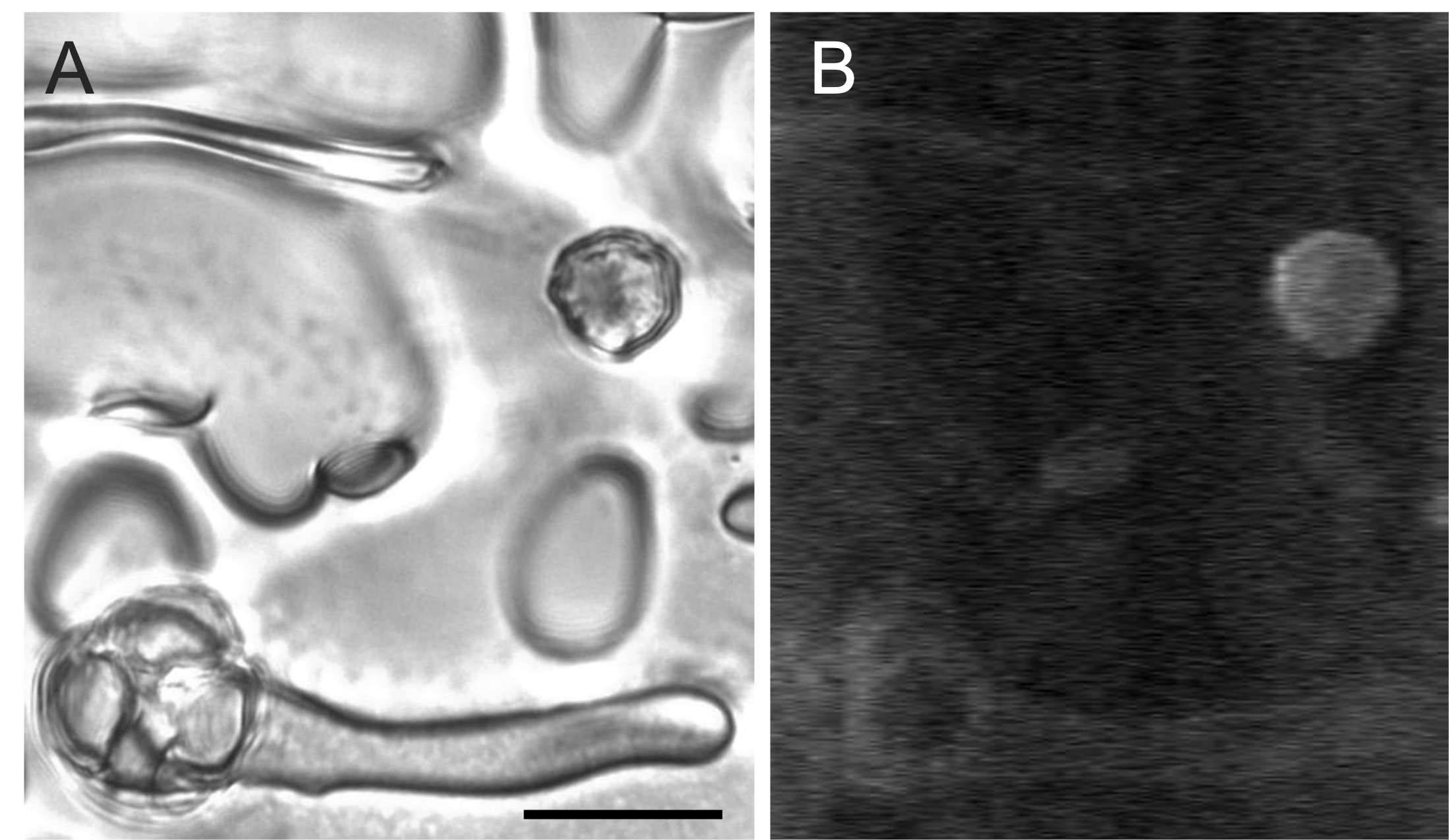

Supplement: Supplementary file 1 [file Image1.JPEG]

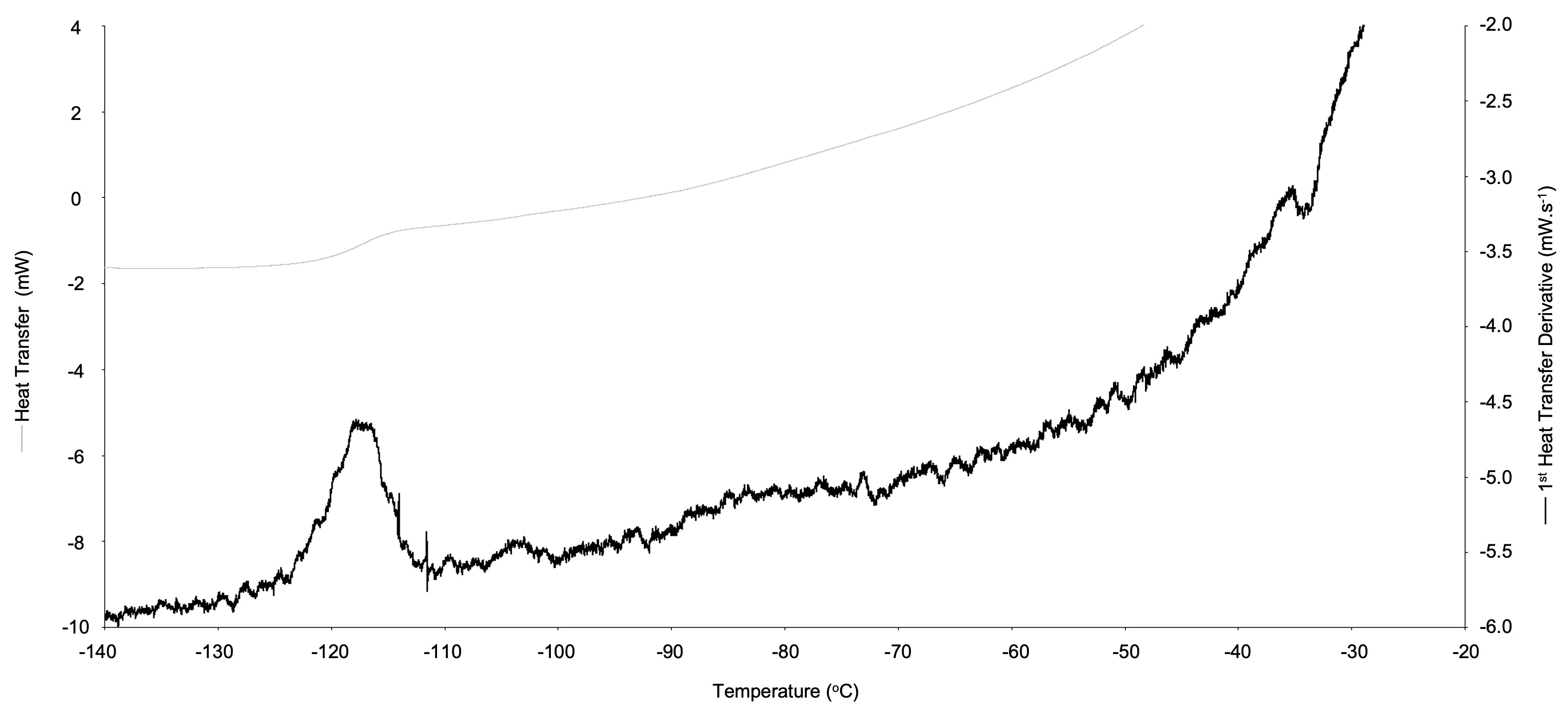

Supplement: Supplementary file 2 [file Image2.JPEG]
